# Emergence of community-associated methicillin-resistant *Staphylococcus aureus* ΨUSA300 among Japanese people with HIV, resulted from stepwise mutations in 2010s

**DOI:** 10.1038/s41598-023-35171-y

**Published:** 2023-05-23

**Authors:** Koh Shinohara, Yuki Uehara, Katsuji Teruya, Takashi Sasaki, Tadashi Baba, Hidemasa Nakaminami, Pegah Kananizadeh, Yuh Morimoto, Yoshimi Kikuchi, Shinichi Oka

**Affiliations:** 1 AIDS Clinical Center, The National Center for Global Health and Medicine, Tokyo, Japan; 2 Department of Clinical Laboratory Medicine, Kyoto University Graduate School of Medicine, Kyoto, Japan; 3 Department of Microbiology, Faculty of Medicine, Juntendo University, Tokyo, Japan; 4 Department of Infectious Diseases, Fujita Health University School of Medicine, 1-98, Dengakugakubo, Kutsukake-cho, Toyoake, Aichi 470-1192 Japan; 5 Animal Research Center, Sapporo Medical University School of Medicine, Sapporo, Japan; 6 Graduate School of Nursing, Seisen Jogakuin College, Nagano, Japan; 7 Department of Microbiology, School of Pharmacy, Tokyo University of Pharmacy and Life Sciences, Tokyo, Japan; 8 Faculty of Health Science, Juntendo University, Tokyo, Japan

**Keywords:** Bacterial infection, Bacterial genomics

## Abstract

Although infection with the methicillin-resistant *Staphylococcus aureus* (MRSA) clone USA300 is extremely rare in Japan, the uniquely evolved clone ΨUSA300 has been reported in Japan. An outbreak of a distinct USA300 clone was recently reported in an HIV/AIDS referral hospital in Tokyo. The present study investigated the evolutionary origin and genetic diversity of USA300-related clones causing regional outbreaks among people living with HIV (PLWHIV) in Tokyo. MRSA isolates collected from PLWHIV in an HIV/AIDS referral center in Tokyo were subjected to whole-genome sequencing and their genetic features were compared with those of previously described USA300 MRSA genomes. Of the 28 MRSAs isolated in 2016–2019, 23 (82.1%) were identified as USA300, with 22 (95.6%) of the latter identified as ΨUSA300. Although the genomic structure of ΨUSA300 was identical to the structures of reference USA300 strains, one clade (cluster A) was found to have acquired 29 previously identified lineage-specific mutations in a stepwise manner. The estimated divergence dates of ΨUSA300 and Cluster A were 2009 and 2012, respectively. These findings suggested that the ΨUSA300 clone had spread among PLWHIVs in Tokyo in the early 2010s, with stepwise acquisition of lineage-specific nonsynonymous mutations.

## Introduction

Community-associated methicillin-resistant *Staphylococcus aureus* (CA-MRSA) infection is a major concern worldwide because of the high burden of the disease^1,2^. The emergence in the late 1990s and early 2000s of a novel USA300 strain of CA-MRSA, which was originally characterized as sequence type (ST) 8 and staphylococcal cassette chromosome *mec* (SCC*mec*) type IV, and possessing Panton-Valentine leucocidin (PVL) and an arginine catabolic mobile element (ACME), markedly altered the epidemiology of skin and soft tissue infections (SSTI) in the USA and other North American countries^1,2^. In contrast with its rapid spread throughout North America and the northern part of South America, USA300 was not prevalent in other parts of the world at that time. Although many individuals in other continents have been infected with the USA300 strain, this strain has failed to become dominant outside the Americas^2–4^.

Until recently, the USA300 strain was responsible only for small sporadic outbreaks in Japan, in both outpatient and nosocomial settings^5–7^; but this strain was not predominant throughout the country^8^. Several recent reports, however, have shown that the prevalence of the USA300 strain has increased among MRSA isolates from patients in Japan with SSTI^9–12^. In addition, several USA300 clones have undergone evolution in Japan, including the USA300-LV/J (ST8-IVc/ACME negative) and ΨUSA300 (with a 12 bp deletion in *ccrB2* on SCC*mec*) strains^13,14^. During the same period, the prevalence of USA300 increased in Korea and Taiwan^15–17^. These findings indicate that the USA300 strain has undergone dissemination in Japan and other Eastern Asian countries.

The outbreak of USA300 among people living with human immunodeficiency virus (HIV) infection (PLWHIV) in Tokyo suggested the local transmission of unique USA300 strains with specific mutations in genes associated with glycolysis, such as *ldh*, *fumC*, *fruA*, *gatC1*, and *nanE*^18^.

To date, however, the genetic relationships among USA300 clones in the eastern Asia have not been fully evaluated.

The aim of the present study was to investigate the evolutionary origin and genetic features of clinical isolates from Japanese PLWHIV. Isolates were subjected to whole-genome sequencing (WGS) and their genetic features were compared with those of previously described USA300 MRSA genomes in Japan and other countries.

## Results

### Description of patients infected with MRSA USA300

A surge of CA-MRSA SSTI was observed in 2014 among PLWHIV in the AIDS Clinical Center (ACC) outpatient clinic in the National Center for Global Health and Medicine (NCGM), Tokyo, Japan. In 2010–2013, MRSA was present in only two (22%) of nine patients with community-onset SSTI from whom *S. aureus* was isolated. However, the prevalence of MRSA sharply increased as 18 (94.7%) of 19 patients with SSTI in 2014–2016. In this study, we collected 28 MRSA isolates from SSTI patients in the ACC outpatient clinic, between August 1, 2016 and January 31, 2019. Of these 28 MRSA isolates, 23 (82.1%) were identified as USA300; ST8, SCC*mec* type IVa, presence of PVL genes, and ACME element and phylogenetic clustering with the USA300 reference genome TCH1516. The flow diagram for the selection of MRSA isolates in this study is shown in Supplementary Fig. 1. The temporal distribution of patients infected with USA300 and non-USA300 isolates is shown in Supplementary Fig. 2. Baseline demographics of the study population are summarised in Supplementary Table 1. All 23 patients infected with USA300 were residents of Japan and male, and 22 patients were men who have sex with men (MSM). Two patients had travelled abroad, to mainland China and Australia, within 3 months before presentation. The median age of these patients was 41 years (range, 25–62 years). Their median CD4 lymphocyte count was 562/µL, with 91% patients having CD4 lymphocyte counts ≥ 200/µL. All patients had received anti-retroviral therapy (ART), and 21 (95%) of 22 patients had HIV viral loads < 50 copies/mL.

The clinical characteristics of SSTI in these patients are shown in Table 1. Subcutaneous abscess was a predominant type of infection. The most common locations of infection were the buttocks and genital regions (48% of patients), with 70% of patients having lesions on their lower bodies. Twelve patients (52%) were admitted to the hospital, but none had bacteremia and there were no fatalities. Nine patients (39%) were initially treated with intravenous antimicrobials, including six with vancomycin and three with beta-lactams; and 10 patients (44%) were initially treated with oral antimicrobials, including six with trimethoprim-sulfamethoxazole, three with beta-lactams, and one with; minocycline. The other four patients (17%) were treated without systemic administration of antimicrobials. Four patients (17%) showed evidence of spontaneous drainage, and five patients (22%) underwent incision and drainage. All 22 patients with outcome data available showed improvement without sequelae; however, two patients had at least two episodes of MRSA SSTI during the study period.

**Table 1:** Clinical characteristics of patients with USA300 methicillin-resistant *Staphylococcus aureus* skin and soft tissue infection.

Characteristic	Patients (%)
**Skin and soft tissue infection diagnosis**	
Abscess	18/23 (78)
Cellulitis	1/23 (4)
Furuncle	3/23 (13)
Erosion	1/23 (4)
**Site of infection (including patients with multiple infection sites)**	
Head, face, neck	5/23 (22)
Trunk	2/23 (9)
Thigh	1/23 (4)
Knee/calf	5/23 (22)
Upper extremities	2/23 (4)
Hand and foot	0/23 (0)
Buttocks, genitals, perineum	11/23 (48)
**Bacteremia**	0/23 (0)
**Hospitalization**	12/23 (52)
**Initial antimicrobials**	
Beta-lactam	6/19 (32)
Clindamycin	0/19 (0)
Macrolide	0/19 (0)
Tetracycline	1/19 (5)
Trimethoprim-sulfamethoxazole	6/19 (32)
Vancomycin	6/19 (32)
**Spontaneous drainage of abscess**	4/23 (17)
**Incision and drainage**	5/23 (22)
**Outcome**	
Relapse	2/22 (9)
Fatal	0/22 (0)

### Genomic characteristics of these USA300 isolates and their relationship with previous isolates from PLWHIVs in Tokyo

**Figure 1:**
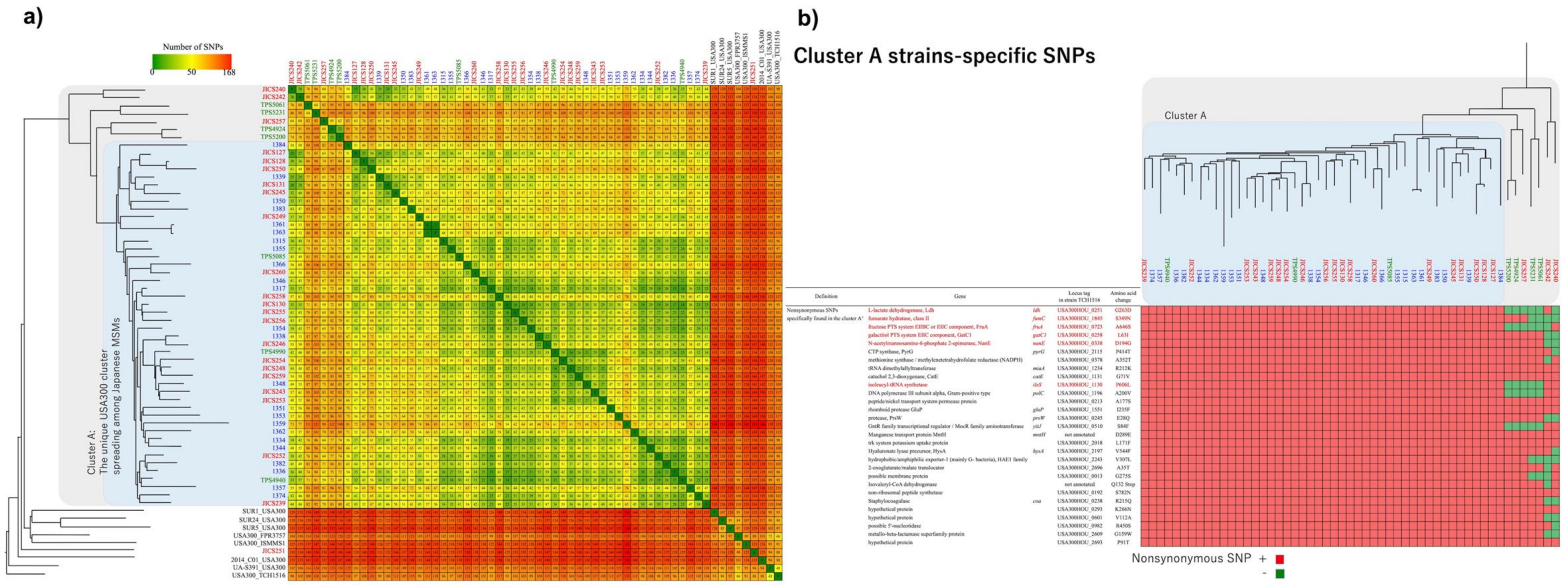
(**a**) Phylogenetic tree of the USA300 strains in this study (red font, n = 23), and previously reported USA300 strains from PLWHIVs in Tokyo (blue font, n = 24), ΨUSA300 reference strains previously isolated from Japanese patients^18,31^ (green font, n = 7) and completely sequenced USA300 strains registered with the National Center for Biotechnology Information (NCBI) (black font, n = 8). SNP analysis showed that 93.90% (2,724,796 of 2,901,719 positions) of the reference genome (strain JICS127) was covered by all strains. SNP differences ranged from 14 to 168. The neighbor-joining tree was constructed by aligning 1450 SNP sites, with strain USA300_TCH1516 used as the outgroup. The numbers of interstrain SNP differences were visualized using a red-yellow-green gradient, with red indicating the highest score (168) and green indicating the lowest score (0). Forty-six isolates were clustered into a single clade (cluster A). (**b**) Heatmap of the 29 nonsynonymous SNPs uniquely found among the isolates in cluster A (highlighted in light blue). The presence and absence of the SNPs were visualized in red and green, respectively. The names, the locus tag in strain TCH1516, and amino acid changes of each nonsynonymous SNP are described in the table. Stepwise acquisition of these 29 SNPs was suspected.

Genomic analysis of the SCC*mec* element showed that 22 of the 23 USA300 isolates from the ACC were positive for the 12 bp deletion in the *ccrB2* gene of the type IV SCC*mec* element^14^ (Supplementary Fig. 1). The relationships of these isolates with previously isolated USA300 isolates were assessed by genomic comparisons and phylogenetic analyses. Specifically, the present isolates were compared with the ΨUSA300 isolates deposited in GenBank under accession No. DRA010484^18^, seven other ΨUSA300 isolates from patients with SSTI in Japan^19^ and eight complete-genome-sequenced USA300 strains registered at NCBI. A phylogenetic tree based on whole genome single nucleotide polymorphism (SNP) is shown in Fig. 1a. Twenty-two (96%) of the 23 strains isolated in the present study, 24 (83%) of the 29 isolates deposited under accession number DRA010484 and all 7 ΨUSA300 isolates were positive for the 12 bp deletion in the *ccrB2* gene and formed a phylogenetic clade. Figure 1b shows a heatmap of the previously reported nonsynonymous SNPs^18^. The ΨUSA300 clade included 46 isolates that formed a cluster containing all of these unique nonsynonymous SNPs (cluster A). A comparison of nonsynonymous SNPs present in isolates inside and outside cluster A suggested that the ΨUSA300 isolates acquired these nonsynonymous SNPs in a multi-phasic manner.

### Phylogenetic and Bayesian comparison of the ΨUSA300 clade with previously reported USA300 isolates

**Figure 2:**
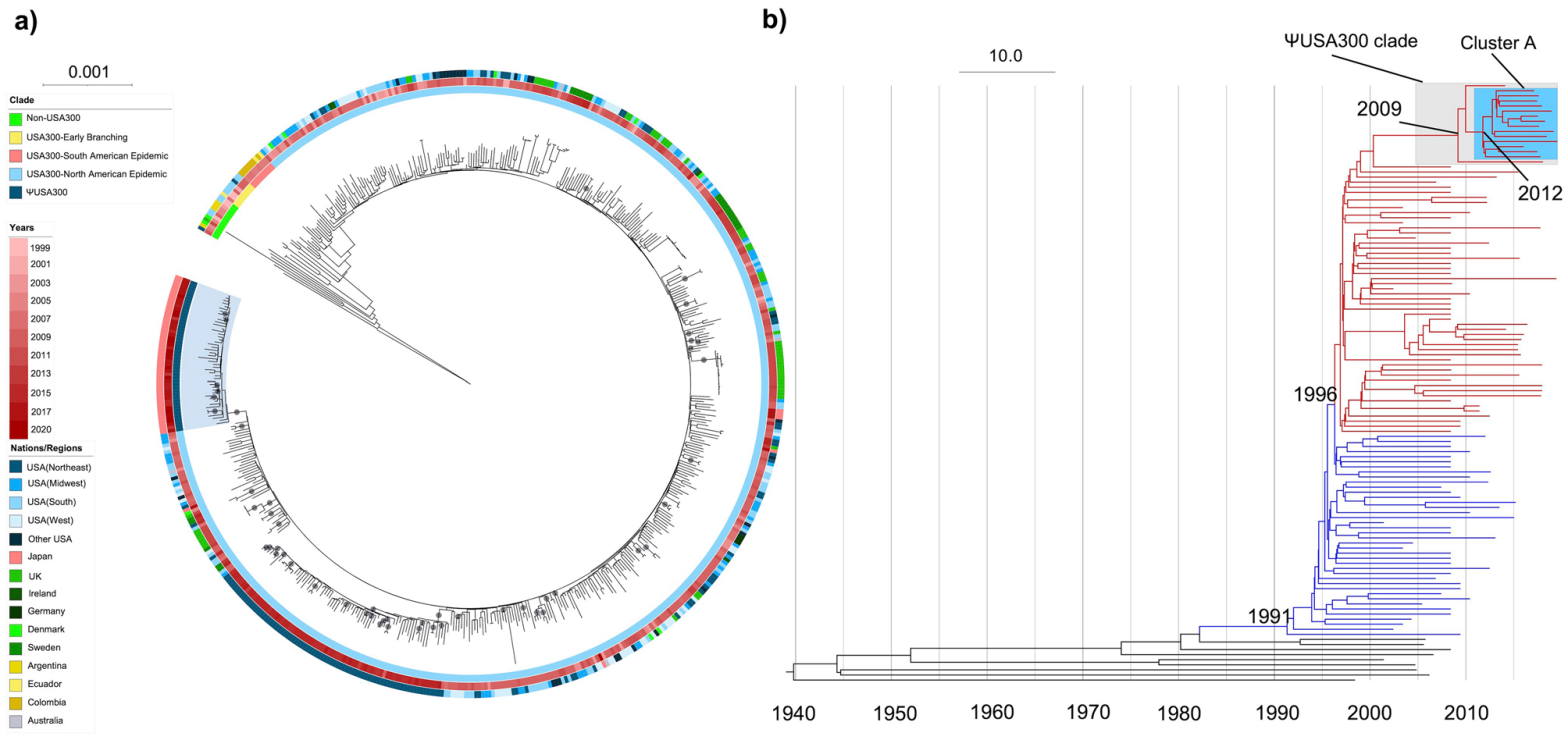
Phylogenetic analyses showing the emergence of the unique USA300 clade in Japan. (**a**) Maximum likelihood (ML) phylogeny of strains of the USA300 lineage. The ML tree was based on 559 genomes and 19,779 concatenated SNPs, rooted relative to the distantly related MSSA strain ERS092996. Gray points represented the bootstrap values 100. The ψUSA300 clade is highlighted in blue. The inner circle indicates the clades of the isolates; the middle circle represents the year of isolation in red, from lightest (1999) to darkest (2020); and the outer circle represents the nations/regions of origin of the isolates. (**b**) Bayesian phylogenetic reconstruction based on 118 isolates. Branches in black are non-USA300 North American epidemic (NAE) isolates, colored according to their fluoroquinolone genotype as susceptible (blue) or resistant (red). ΨUSA300 clade branches are highlighted in gray and cluster A branches are highlighted in blue. The predicted dates of divergence of USA300 are indicated.

The genomic relationships between the ΨUSA300 isolates and other USA300 isolates were assessed by phylogenetic analysis of 559 sequence type (ST) 8 isolates. The maximum-likelihood tree is shown in Fig. 2a. The ΨUSA300 isolates formed a unique clade genetically distant from the other USA300 isolates. The phylogenetic tree that included the USA300 strains isolated from patients in Taiwan is shown in Supplementary Fig. 3. The Taiwanese USA300 strains formed four major clades, each consisting of more than four strains. These four clades were genetically distinct from the ΨUSA300 clade. Two of the four clades included non-ΨUSA300 Japanese USA300 strains, one clade included three Japanese isolates and the fourth clade included one Japanese isolate.

The time of divergence of the ΨUSA300 isolates was estimated by a Bayesian approach using BEAST2, with representatives of all phylogenetic subclades in the parent phylogeny included in the analyzed subset. The years of divergence of the USA300 North American epidemic (USA300-NAE) clone and its fluoroquinolone-resistant subclade were estimated to be around 1991 (95% highest posterior density [HPD]: 1989–1994) and 1996 (95% HPD: 1995–1998), respectively, estimates in good agreement with previous studies^20,21^. The years of divergence of the ΨUSA300 clone and cluster A were estimated to around 2009 (95% HPD: 2008–2011) and 2012 (95% HPD: 2011–2013), respectively (Fig. 2b).

### Antimicrobial susceptibility of the USA300 isolates and their associated resistance genes

The results of antimicrobial susceptibility tests of the 23 USA300 isolates in the present study are summarized in Supplementary Table 2. Of the 22 ΨUSA300 isolates, 19 were included in cluster A. All USA300 isolates were phenotypically susceptible to vancomycin, linezolid and minocycline, but resistant to levofloxacin and erythromycin. Two (9%) of the 23 erythromycin-resistant isolates were positive for inducible clindamycin resistance. Genotypically, all 23 isolates had *msr*A, and the two isolates with inducible clindamycin resistance had *erm*C. Assessments of fluoroquinolone resistance genotypes showed that all 23 isolates possessed the *gyr*A 84L and *grl*A 80Y mutations. Four (17%) of the 23 isolates were phenotypically resistant to gentamicin and possessed the *aacA-aphD* gene. Thus, the antimicrobial resistance patterns of ΨUSA300 isolates belonging to cluster A did not differ significantly from those of USA300 isolates outside cluster A.

### Complete genome sequence and genomic characteristics of the ΨUSA300 strain JICS127

**Figure 3:**
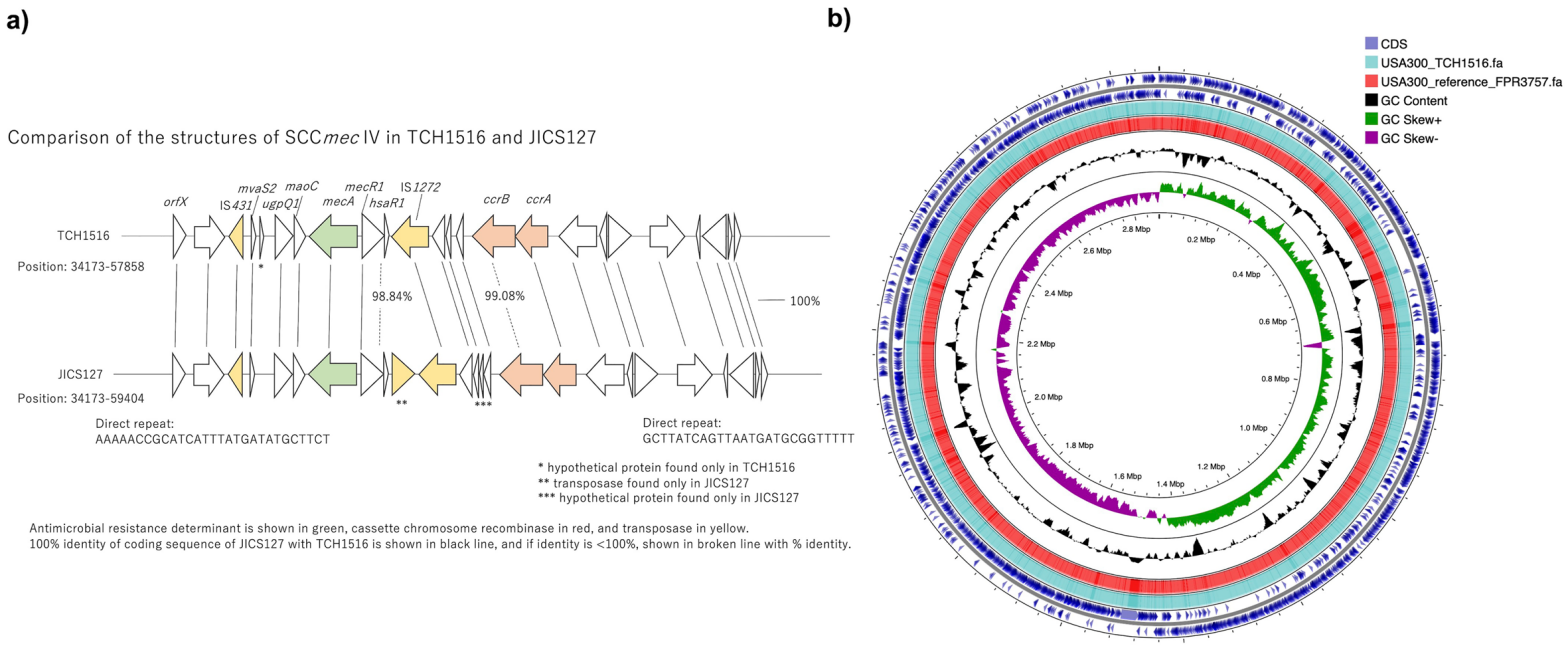
Comparison of the genomic structures of JICS127 and reference USA300 strains. (**a**) Genomic structures of SCC*mec* IV in TCH1516 (top) and JICS127 (bottom). Antimicrobial resistant determinants are shown in green, cassette chromosome recombinases in red, and transposases in yellow. Solid black lines represent 100% identity of the coding sequences of JICS127 and USA300_TCH1516, and broken lines represent < 100% identity, with the % identity indicated. (**b**) Comparative circular map of *Staphylococcus aureus* USA300_TCH1516 (NC_010079) and USA300_FPR3757 (NC_007793) to ΨUSA300_JICS127. The circles display the following information (from outside to inside): CDSs of ΨUSA300_JICS127 on the + and − strands, blastn results of USA300_TCH1516 and USA300_FPR3757, GC contents of ΨUSA300_JICS127, GC positive skewing (green) and GC negative skewing (violet) of ΨUSA300_JICS127.

The chromosome of the ΨUSA300 JICS127 isolate was 2,879,025 bp long and contained 2,805 genes^22^. A comparison of the structures of SCC*mec* IV in USA300_TCH1516 and JICS127 (Fig. 3a) showed that their overall genomic structures were preserved. A comparison of the circular maps of JICS127 with the two USA300 reference strains showed that the repertoire of the coding sequence (CDS) and the global genetic structure of JICS127 were in excellent agreement with those of USA300_TCH1516 and USA300_FPR3757 (Fig. 3b).

## Discussion

The USA300 clone of CA-MRSA has become a major health burden in both community and hospital settings worldwide. Although hitherto it is extremely rare in Japan, multiple USA300-like clones, such as USA300-LV/J and ΨUSA300, have been detected recently^13,14^. Genomic analysis in the present study revealed that most of the recent CA-MRSA isolates from PLWHIVs in Tokyo were identified as ΨUSA300. ΨUSA300 had evolved from an original USA300 clone in Japan around 2009 and underwent additional evolution among PLWHIVs in Tokyo around 2012 through the stepwise acquisition of lineage-specific nonsynonymous mutations. To our knowledge, this is the first report to assess the epidemiology and process of evolutionary success of ΨUSA300 generated among Japanese PLWHIVs.

Although ΨUSA300 has become the predominant USA300 clone among PLWHIVs in Tokyo, ΨUSA300 has not been detected to date in other countries. Genomic analysis showed that ΨUSA300 was comparable to USA300 clones isolated from patients at another hospital in Tokyo. Moreover, all cases in the present study were community-onset, and 22 of 23 patients infected with USA300 had no recent history of hospitalization. These findings suggest that the community spread of ΨUSA300 has recently become more serious in Tokyo. This ΨUSA300 clone was first described in 2018 as an ST8, USA300-like strain, positive for PVL and ACME, but could not be identified as SCC*mec* type IV by conventional multiplex PCR, due to a 12 bp deletion in *ccrB2*^14^. The present study found that the entire genomic structure of the ΨUSA300 JICS127 strain was almost identical to the structures of the USA300 TCH1516 and RFP3757 strains, with pairwise SNP analysis showing that the number of SNPs ranged from 97 to 132. However, the ΨUSA300 variants isolated from PLWHIVs in the present study shared the lineage-specific nonsynonymous mutations in metabolic genes associated with glycolysis, such as *ldh*, *fumC*, *fruA*, *gatC1*, and *nanE*^18^. These mutations, which enhance glycolysis, have been reported to contribute to the adaptation of these strains to human skin^23^.

The finding, that antimicrobial resistance patterns did not differ among ΨUSA300 and other USA300 strains, suggests that the successful spread of ΨUSA300 strains among the Japanese MSM community was not due to selective pressure by antibiotics but to nonsynonymous mutations that enhanced the ability of these strains to colonize the skin.

Recent reports from Korea and Taiwan have also suggested that the prevalence of USA300 clones has increased^15–17^. Although whole genome sequences of the Korean isolates have not been determined, the isolates from Taiwan consisted of multiple USA300 clones similar to European and American types, but were not associated with ΨUSA300. Analysis showed that the non-ΨUSA300 clade was mixed with Taiwanese and Japanese USA300 strains in a phylogenic tree, suggesting the transmission of USA300 strains among Eastern Asian countries.

The time of emergence of ΨUSA300 variants was assessed using Bayesian methods, with results suggesting that ΨUSA300 had diverged from the original USA300 strains around 2009. Strains belonging to cluster A, which had acquired all the lineage-specific nonsynonymous SNPs, have been endemic among PLWHIVs in Tokyo since early 2010s. Despite the evolutionary success of USA300 in North America, the USA300 clone has not yet been successfully established in other continents^3,4,24^. Moreover, its prevalence has tended to decrease over time^2^, even at some locations in the United States^25^. Whole-genome population genetic analysis of isolates from North and South America, Europe, and Australia suggested that the effective population size of the USA300 lineage declined sharply after 2008^3^. In contrast, the prevalence of ΨUSA300, which evolved from Euro-American USA300, has increased in Japan during the last decade.

As in other countries, antimicrobial stewardship has been evolving in Japan. The goals of the Japanese national action plan for AMR include 50% reductions in the amounts of oral cephalosporins, macrolides and fluoroquinolones administered in 2020 compared with 2013^26^. Changing trends in antimicrobial prescriptions may prevent the dissemination of multi-drug resistant ΨUSA300 strains. Although ΨUSA300 strains have not been isolated to date from patients in countries outside Japan, studies should focus on the global dissemination of these clones.

To date, there have been no nation-wide studies of ΨUSA300 in Japan, suggesting the need for systematic WGS-based surveillance to understand the epidemiology of ΨUSA300 strains in Japan. SSTI due to infection with USA300 or PVL-positive MRSA has also emerged in PLWHIVs outside Tokyo^27,28^. Concerns have also arisen regarding the invasion of ΨUSA300 strains into hospitals. Surveillance in both community and nosocomial settings is needed to evaluate the degree of infiltration of ΨUSA300 strains. In addition, further studies are needed to determine the relationship between the 12 bp deletion in *ccrB* and the acquired nonsynonymous SNPs of strains in the ΨUSA300 clade with their virulence.

In conclusion, the present study revealed that the regional outbreak of SSTI among PLWHIVs in Tokyo was caused by infection with the ΨUSA300 clade. This clade evolved and emerged in Japan in the early 2010s after the multiphasic acquisition of unique nonsynonymous SNPs. Effective infection control and prevention, as well as appropriate antimicrobial stewardship, require surveillance utilising the WGS approach to monitor the trend of USA300 lineages in Japan.

## Methods

This study was conducted in the ACC, NCGM, Tokyo, Japan, from August 2016 to January 2019. We identified all SSTI patients from whom MRSA was isolated in the ACC outpatient clinic in the study period. Patients infected with HIV were identified by review of their electronic medical records, with HIV patients aged ≥ 18 years considered eligible for inclusion in this study.

### Collection of patient data

The electronic medical records of consecutive patients with SSTI from August 2016 to January 2019 and positive for MRSA cultured from infection sites were retrospectively reviewed. Factors recorded included patient demographic characteristics (e.g., date of birth, sex, race/ethnicity), dates of clinic visits, SSTI presentation, CD4+ lymphocyte count, HIV viral load, status of ART, and SSTI treatment and outcome. A diagnosis of SSTI was confirmed by review of clinician notes and prescription data in patients’ medical records. Post-operative infections and infections of prostheses or medical devices were excluded. Infection with CA-MRSA was defined as previously described^29^.

### Microbiological evaluation

All clinical isolates were identified as *S. aureus* by Gram-positive, cluster-forming morphology by Gram staining, proliferation on mannitol salt agar, and coagulase production tests. Susceptibility to commonly used antimicrobial agents was determined using the broth microdilution method with the dry plate DP32 (Eiken Chemical Co., LTD., Tokyo, Japan). Antimicrobial susceptibility results were interpreted using the guidelines of the Clinical and Laboratory Standards Institute^30^.

### Genome sequencing, alignment, assembly and genetic characterization

Genomic DNA was extracted using QIAamp DNA Mini Kits (QIAGEN, Hilden, Germany), according to the manufacturer’s instructions. Samples were prepared for WGS using Nextra XT DNA sample preparation kits (Illumina Inc., San Diego, CA, USA), and sequencing was performed using a MiSeq sequencer (Illumina Inc.). Sequenced reads were assembled with Unicycler v0.4.8^31^. Multilocus sequence types were identified from the assembled contigs using a BLAST search of the *S. aureus* MLST database (available at: https://pubmlst.org/organisms/staphylococcus-aureus/), with results assigned to clonal complexes (CCs). SCC*mec* types, virulence genes, and antimicrobial resistant genes were determined using SCC*mec*Finder^32–35^, VirulenceFinder^36^, and ResFinder^37^ respectively, on the website of the Center for Genomic Epidemiology (http://www.genomicepidemiology.org). CC8-SCC*mec* IVa-PVL gene-positive isolates were identified and formed the basis for the remaining genetic analyses. Sequence reads of USA300 strains from the United States, South American countries (Argentina, Colombia, Ecuador), European countries (Denmark, Germany, Ireland, Sweden, and the United Kingdom), Australia, and Japan^3,4,18,20,38^ were downloaded from the European Nucleotide Archive (ENA) website (https://www.ebi.ac.uk/ena) or obtain directly from the author^19^. Paired-end reads were aligned to the *S. aureus* USA300_TCH1516 reference genome (GenBank accession no. GCA_000017085.1) using the Burrows–Wheeler Aligner tool (BWA) v0.7.17^39^, after removing the duplicate reads using SAMtools v1.12^40^. Aligned reads were sorted and indexed using SAMtools and the Genome Analysis Toolkit (GATK, v4.1.9) Picard^41^. SNP calling was performed with the GATK HaplotypeCaller according to the best practices of the Broad Institute. Candidate SNPs in all retained genomes were filtered with a base quality of ≥ Q30 and a read depth ≥ 10. The molecular epidemiology of the USA300 strains, including the USA300 isolates from Taiwan, was investigated using the contig data^16,17^ downloaded from the ENA website. Sequences were aligned to the reference strain USA300_TCH1516 and variants of the contigs using Snippy v4.6.0^42^.

### Complete genome sequencing of the ΨUSA300 strain, JICS127

The genome of the ΨUSA300 strain JICS127 was sequenced using a PacBio RS II sequencer (Pacific Biosciences of California, Inc., Menlo Park, CA, USA) and an Illumina Miseq (Illumina Inc.), and assembled using HGAP3 (PacBio DevNet; Pacific Biosciences of California, Inc.), as described^22^. De novo assembly produced two circular contigs composed of a chromosome and a plasmid. Genes were annotated on the Rapid Annotations using Subsystems Technology (RAST) server^43^.

### Phylogenetic analyses and inferring divergence time using genome-wide data sets

The relationships among the 23 USA300 strains isolated in the present study, 24 other USA300 strains isolated from PLWHIVs in Tokyo during the same period, seven ΨUSA300 reference strains isolated from Japanese patients^18,19^ and eight completely sequenced strains registered with the National Center for Biotechnology Information (NCBI) were evaluated by phylogenetic tree analysis. A neighbor-joining tree was constructed by alignment of SNP sites. Strain USA300_TCH1516 was used as the outgroup.

The relationships among USA300 and closely-related ST8 *S. aureus* strains from other countries were also assessed by phylogenetic analysis of 559 CC8 *S. aureus* genomes, including methicillin-susceptible *S. aureus* (MSSA), non-USA300 MRSA, USA300 early branching (EB), USA300 South American epidemic (SAE) and USA300 North American epidemic (NAE) MRSA clones^44^. After removing the suspected recombination sites using Gubbins v2.4.1^45^, 19,779 high-confidence variable positions were used for the analysis, with the distantly related MSSA strain ERS092996 defined as the outgroup^3^. A maximum-likelihood phylogenetic tree was generated using RAxML-NG v1.0.3^46^ and a GTR + Г substitution model. The nodes were supported by 100 bootstrap replicates.

The relationships between Japanese and Taiwanese USA300 isolates were also investigated by phylogenetic analysis using contig data of the 640 isolates, including 559 CC8 isolates and 81 Taiwanese USA300 isolates. SNP alignments without predicted recombination sites were constructed by Snippy and Gubbins, and a maximum-likelihood phylogeny was generated using RAxML-NG and a GTR + Г substitution model.

Time of strain divergence was analyzed in a subset of 118 isolates with the Bayesian Markov chain Monte Carlo framework, using the BEAST2 v2.6.6 package^47^, with recombination predicted using Gubbins. Predicted recombination sites were removed, with the final SNP alignments used as the input dataset for BEAST2. The tree was calibrated using the sampling dates of the isolates from 1999 and 2020. A strict, exponential-relaxed, and lognormal-relaxed molecular clock with constant size coalescent and Bayesian skyline coalescent was used. For each model, BEAST2 was run for 200 million generations, sampling every 10,000 states, using the GTR + Г substitution model. Convergence and mixing were checked with Tracer v1.7.2. The best fit model was chosen based on the nested sampling^48^. A strict molecular clock and a constant-size coalescent model were chosen. The phylogenetic trees were visualized using FigTree v1.4.4 (available at https://github.com/rambaut/figtree/) and iTOL v6^49^.

### Ethics declaration

Informed consent from the study participants was waived by the National Center for Global Health and Medicine because of the retrospective design of this study and use of stored bacterial samples. The study protocol was approved by Human Research Ethics Committee of the National Center for Global Health and Medicine (Approved No. NCGM-G-003353-00). The study accorded with the principles of the Declaration of Helsinki.

## Supplementary Information

Supplementary Information 1.

Supplementary Information 2.

Supplementary Information 3.

## Data Availability

The read data of WGS of the isolates obtained in this study have been deposited in DDBJ under accession number DRR227240-DRR227267. The complete genome data of JICS127 has been deposited in DDBJ under accession numbers AP025693, AP025694 and AP025695. All other relevant data are within the paper and in Supplementary Dataset 1 and 2.
