# Supplementary material for: Emergence of community-associated methicillin-resistant Staphylococcus aureus ΨUSA300 among Japanese people with HIV, resulted from stepwise mutations in 2010s
Source: Sci Rep. 2023 May 23;13:8322. doi: 10.1038/s41598-023-35171-y (PMC10205742; doi:10.1038/s41598-023-35171-y)

Supplementary Materials

Supplementary Table 1. Demographic and epidemiological characteristics of the patients with USA300 methicillin-resistant *Staphylococcus aureus* skin and soft tissue infection.

Characteristic	Patients (%), N=23
Age in year, median (range)	41 (25-62)
Sex	
Male, n (%)	23/23 (100)
Female, n (%)	0/23 (0)
Race	
Asian, n (%)	23/23 (100)
Non-Asian	0/23 (0)
Residence	
Japan	23/23 (100)
Men who have sex with men	22/23 (96)
Illicit drug use	2/23 (9)
Foreign travel in past 12 months	2/23 (9)
CD4+ lymphocyte count (/μL)	
>500	14/22 (64)
200-500	6/22 (27)
<200	2/22 (9)
HIV* viral load (/mL)	
>1,000	0/22 (0)
50 – 1,000	1/22 (5)
20 - 50	5/22 (23)
<20	17/22 (77)
Receipt of antiretroviral therapy	23/23 (100)
Hospitalization in past 12 months	1/23 (5)
Antimicrobial use in past 12 months	2/23 (9)

* HIV: human immunodeficiency virus

Supplementary Table 2. Minimum inhibitory concentrations (MICs) of antimicrobial agents against USA300 isolates.

Antimicrobial agents[a]	MICs (mg/L)							
	ψUSA300 MRSA strains belonged to the Cluster A (n=19)				non-Cluster A USA300 MRSA strains (n=4)			
	Range	MIC^{b}_{50}	MIC^{b}_{90}	No. of susceptible strains (%)	Range	MIC_{50}	MIC_{90}	No. of susceptible strains (%)
Oxacillin	>4	>4	>4	0 (0)	>4	>4	>4	0 (0)
Cefoxitin	>16	>16	>16	0 (0)	>16	>16	>16	0 (0)
Ampicillin	16->16	>16	>16	0 (0)	8->16	8	>16	0 (0)
Cefazolin	>16	>16	>16	0 (0)	8->16	>16	>16	1 (25.0)
Cefmetazole	16-32	32	32	9 (47.4)	8-32	16	32	2 (50.0)
Flomoxef[c]	16->16	16	>16	-	4->16	16	>16	-
Imipenem	2->8	2	>8	10 (52.6)	0.5->8	≤0.25	2	3 (75.0)
Gentamicin	0.5->8	1	>8	13 (68.4)	≤0.25->8	1	>8	3 (75.0)
Arbekacin[c]	≤0.5-8	2	>8	16 (84.2)	≤0.5-2	2	2	4 (100)
Minocycline	≤2	≤2	≤2	19 (100)	≤2	≤2	≤2	4 (100)
Erythromycin	>4	>4	>4	0 (0)	>4	>4	>4	0 (0)
Clindamycin	≤0.25->2	0.12	>2	18 (94.7)	≤0.25->2	0.25	>2	3 (75.0)
Levofloxacin	>4	>4	>4	0 (0)	>4	>4	>4	0 (0)
Vancomycin	1-2	1	2	19 (100)	1-2	1	2	4 (100)
Teicoplanin	≤0.5	≤0.5	≤0.5	19 (100)	≤0.5-1	≤0.5	1	4 (100)
Linezolid	2-4	4	4	28 (100)	4	4	4	4 (100)
Fosfomycin[c]	≤32	≤32	≤32	-	≤32	≤32	≤32	-
Sulfamethoxazole/ trimethoprim	≤9.5/0.5- >38/2	≤9.5/0.5	>38/2	18 (94.7)	≤9.5/0.5	≤9.5/0.5	≤9.5/0.5	4 (100)

[a] MICs of oxacilllin and cefoxitin were determined by the agar dilution method; all other MICs were determined by the broth microdilution method.

[b] MIC_{50}/MIC_{90}, MIC required to inhibit the growth of 50% or 90% of the strains, respectively.

[c] For arbekacin, Cliniral Laboratory Standards Institute (CLSI) breakpoint of gentamicin is used as a substitute; For flomoxef and fosfomycin, no breakpoints are determined by CLSI.

Supplementary Figure 1. Flow diagram of the selection of the methicillin-resistant *Staphylococcus aureus* (MRSA) isolates in this study.

ST, sequence type. SCC*mec*, staphylococcal chromosomal cassettes *mec*. PVL, Panton-Valentine leucocidin. ACME, arginine catabolic mobile element.

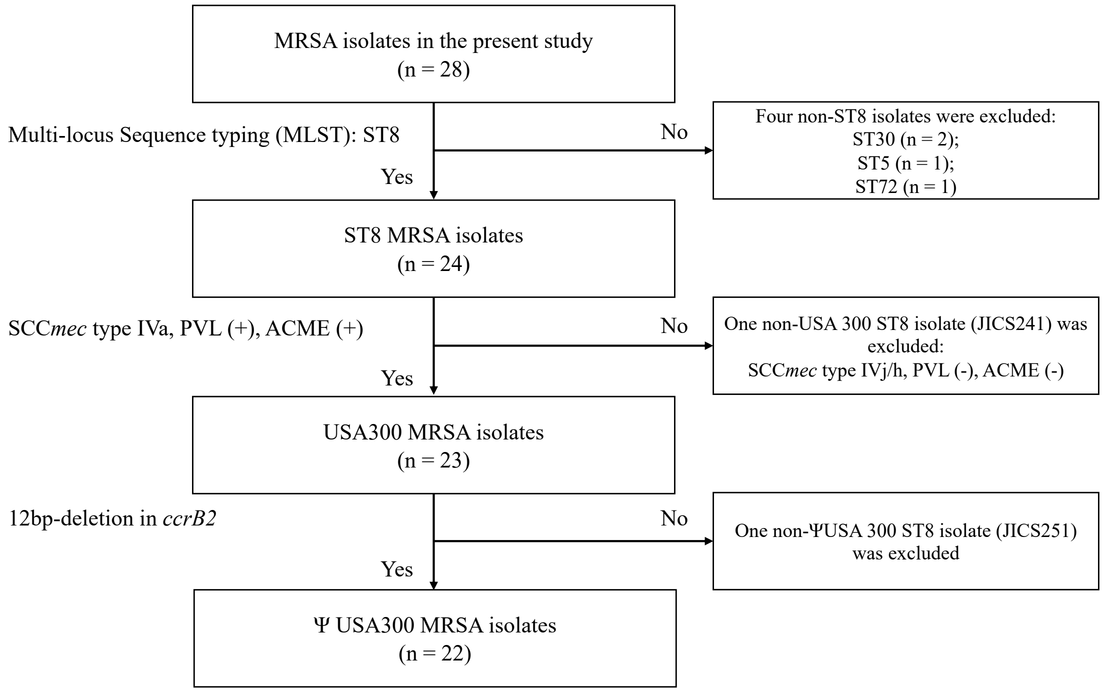

Supplementary Figure 2. Temporal distribution of the case with skin and soft tissue infection caused by methicillin-resistant *Staphylococcus aureus*, according to USA300 strains or not.

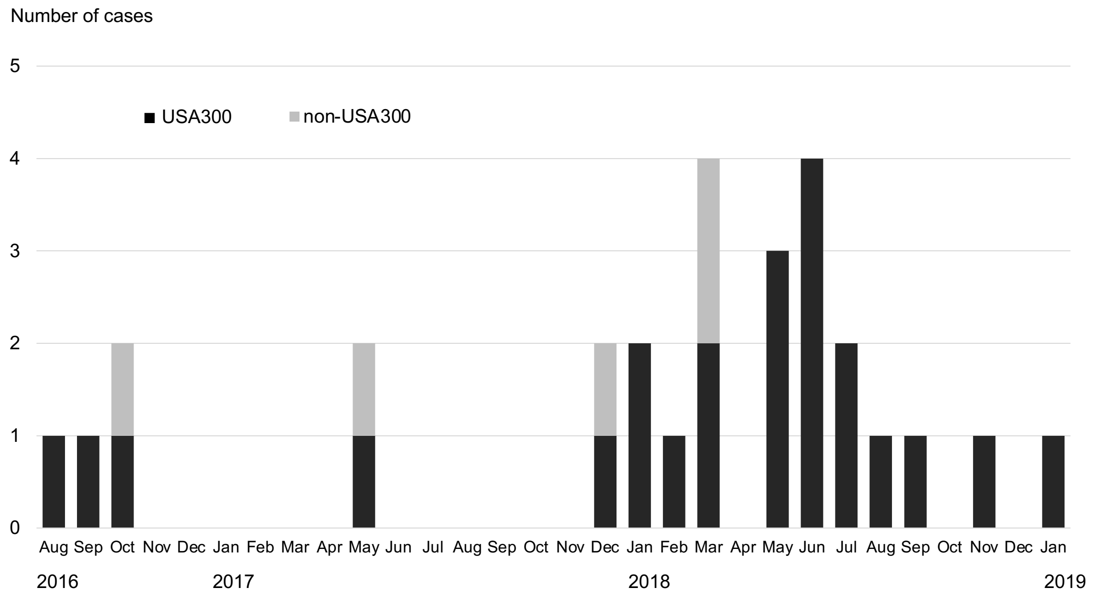

Supplementary Figure 3. Maximum Likelihood phylogeny of USA300 lineage, including Taiwan isolates. ML tree based on 640 genomes and a total of 9,625 SNPs. Outer circle represents the nations/regions isolated. Branches including only Japanese isolates, only Taiwanese isolates, Japanese and Taiwanese isolates were highlighted in blue, grey, and orange, respectively.

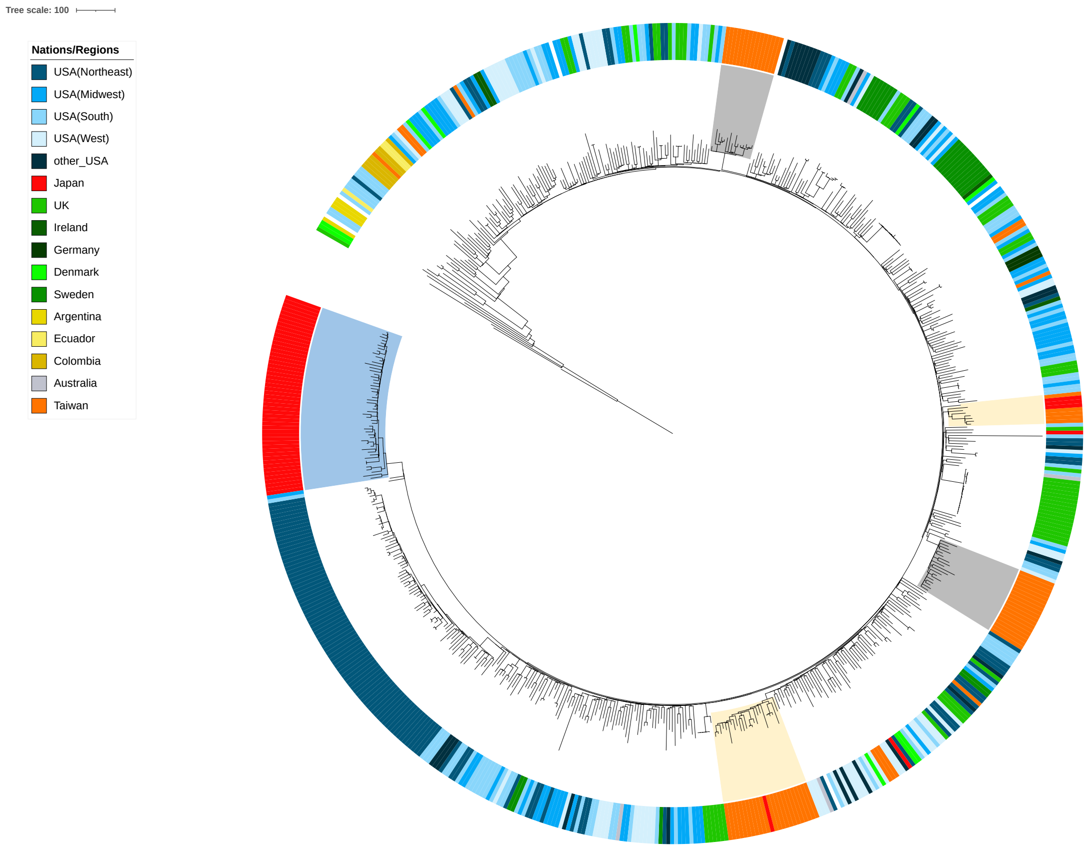

Supplement: Supplementary file 3 — Supplementary Information 3. [file 41598_2023_35171_MOESM3_ESM.pdf]
